# Stenting the Eustachian tube to treat chronic otitis media - a feasibility study in sheep

**DOI:** 10.1186/s13005-018-0165-5

**Published:** 2018-05-04

**Authors:** Friederike Pohl, Robert A. Schuon, Felicitas Miller, Andreas Kampmann, Eva Bültmann, Christian Hartmann, Thomas Lenarz, Gerrit Paasche

**Affiliations:** 1 Department of Otolaryngology, Hannover Medical School, Carl-Neuberg-Str. 1, 30625 Hannover, Germany; 2 Hearing4all Cluster of Excellence, Hannover Medical School, Hannover, Germany; 3 Clinic for Cranio-Maxillo-Facial Surgery, Hannover Medical School, Hannover, Germany; 4 Institute of Diagnostic and Interventional Neuroradiology, Hannover Medical School, Hannover, Germany; 5 Department of Neuropathology, Hannover Medical School, Hannover, Germany

**Keywords:** Auditory tube, Middle ear ventilation, Stent, Otitis media, Sheep as animal model, Tissue reaction

## Abstract

**Background:**

Untreated chronic otitis media severely impairs quality of life in affected individuals. Local destruction of the middle ear and subsequent loss of hearing are common sequelae, and currently available treatments provide limited relief. Therefore, the objectives of this study were to evaluate the feasibility of the insertion of a coronary stent from the nasopharynx into the Eustachian tube in-vivo in sheep and to make an initial assessment of its positional stability, tolerance by the animal, and possible tissue reactions.

**Methods:**

Bilateral implantation of bare metal cobalt-chrome coronary stents of two sizes was performed endoscopically in three healthy blackface sheep using a nasopharyngeal approach. The postoperative observation period was three months.

**Results:**

Stent implantation into the Eustachian tube was feasible with no intra- or post-operative complications. Health status of the sheep was unaffected. All stents preserved their cylindrical shape. All shorter stents remained in position and ventilated the middle ear even when partially filled with secretion or tissue. One of the long stents became dislocated toward the nasopharynx. Both of the others remained fixed at the isthmus but appeared to be blocked by tissue or secretion. Tissue overgrowth on top of the struts of all stents resulted in closure of the tissue-lumen interface.

**Conclusion:**

Stenting of the Eustachian tube was successfully transferred from cadaver studies to an in-vivo application without complications. The stent was well tolerated, the middle ears were ventilated, and clearance of the auditory tube appeared possible. For fixation, it seems to be sufficient to place it only in the cartilaginous part of the Eustachian tube.

**Electronic supplementary material:**

The online version of this article (10.1186/s13005-018-0165-5) contains supplementary material, which is available to authorized users.

## Background

Acute and chronic otitis media continue to be significant issues in human medicine [1]. Especially otitis media with effusion (OME), which is not only common in children under the age of 10, but also the most prevalent reason why advice and treatment from an otorhinolaryngology specialist are needed [2]. In approximately 20% of patients, symptoms become chronic [2], causing severe and often irreversible damage to middle ear structures, including the tympanic membrane and ossicular chain. In the development of these diseases, the Eustachian (or auditory) tube (ET) is one of the key factors [2, 3].

The ET forms the only connection between the middle ear and the nasopharynx. It consists of an inelastic bony part that begins at the protympanum of the middle ear and covers one third of the ET’s full length in humans. This merges into an elastic cartilaginous part, which extends over the remaining two thirds and ends in the nasopharynx. The conjunction of both parts creates a narrow passage, the isthmus [4]. The most important functions of the ET are the transport of secretion, middle ear ventilation, and protection against pathogenic microorganisms [3], but also protection from nasopharyngeal sound and reflux [5]. If one or more of these functions cannot be maintained, this can lead to a dysfunctional Eustachian tube (ETD) and middle ear effusion, followed by middle ear inflammation.

To treat ETD and OME, several different approaches, such as a PVC tube with attached thread inserted via the perforated tympanic membrane [6], a Silastic® tube, inserted via the tympanic orifice of the ET [7], or a gold wire, remaining in-situ for years, with rare rejection and initially good functions [8] have been applied in patients but with limited long-term success [9, 10]. Current management includes conservative methods like the Valsalva maneuver for pressure equalization; nasal douching with saline solution, or nasal application of decongestants, antihistamines, or corticosteroids. The most common surgical approach is the insertion of a tympanostomy tube into the tympanic membrane [11].

Apart from this surgery, two more approaches are used: Eustachian laser tuboplasty, in which enlarged mucous membranes and cartilage are removed to avoid obstruction [12], and balloon dilatation, in which a balloon catheter is inserted into the cartilaginous part of the ET and inflated to loosen adhesions and dilate the lumen [13]. Additionally, the topical application of fluids directly into the ET [14] has recently emerged. Despite the fact that these numerous methods appear in practical use and literature, according to Llewellyn et al. [11], there is little consensus about indications of treatment and moreover, conclusions regarding efficacy have been questioned. In addition, the causes of dysfunction and the mechanisms of intervention and long-term clinical outcomes need to be fully assessed [5].

In preclinical research in chinchilla and rabbit, a poly-L-lactide ET stent, not adapted to the size of the animal, was implanted through the bulla and tympanic membrane and investigated, with moderate outcome [15, 16]. The sheep was also evaluated for preclinical assessment of middle and inner ear implants [17], and the feasibility of endoscopic implantation of a commercially available coronary stent through the nasopharyngeal orifice of the ET was proven in a cadaver study [18], in order to test stents sized for human application in a large animal model. To investigate the feasibility of this approach in-vivo, in the present study differently sized coronary stents were implanted into the ETs of blackface sheep.

In addition, it would be beneficial to have an adequate disease model available. There are models described in the literature using either cauterization of the ET, knock-out mice, or Streptococcus pneumonia in the rat [19, 20]. In chinchilla, aseptically triggered OME was induced [21] with inflammatory mediators, platelet activating factor (PAF) [22], and Prostaglandin E_2_ [21], which provides an easily applied, reversible method, without the risk of uncontrolled infection of the animal.

Therefore, the objectives of the current study were to evaluate the feasibility of in-vivo insertion of a commercially available coronary stent from the nasopharynx into the ET, and to analyze how such stents are tolerated over the course of three months. Additionally, induced aseptic otitis media was evaluated as a potential disease model.

## Methods

### Ethics approval

The State Office for Consumer Protection and Food Safety, Dept. of Animal Welfare, in accordance with the German and European animal welfare legislation, approved this study under number 12/1089. With regard to the valid directives for accommodation, care, and usage of experimental animals, the sheep were cared for, and the experiments were performed in a central animal facility.

### Stents

ProKinetik Energy® stents (Biotronik, Berlin, Germany) consisting of a non-degradable cobalt-chrome alloy, with a strut thickness of 60 μm and a recoil of less than 5 %, were implanted in two different sizes: 2.75 mm × 26 mm (left ear) and 2.0 mm × 20 mm (right ear). Stents were mounted on an expandable catheter for insertion (Rapid exchange catheter, length: 1.4 m).

### Animals and study design

**Fig. 1:**
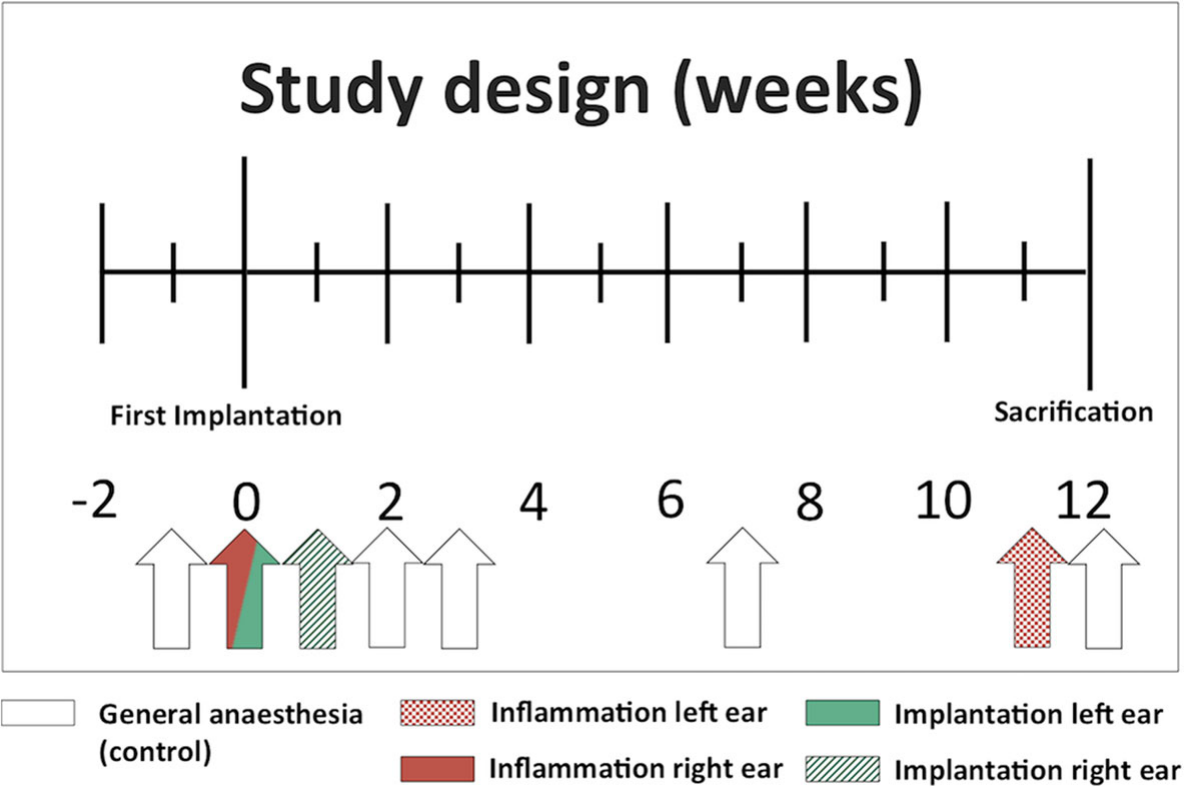
Study design in weeks. The arrows indicate time points of general anesthesia with endoscopic control (white), instillation of inflammatory mediators, or stent implantation

ETs of three healthy adult (2 to 4 years) female blackface sheep were stented bilaterally, with a delay of one week between sides. One week prior to the first implantation, an initial bilateral control of external and middle ears and endoscopic examination of the nasopharyngeal orifices of the ET was performed under general anesthesia. At the time of the first implantation, a sterile middle ear inflammation was triggered in the second ear followed by implantation of the second stent one week later. Regular endoscopic examinations of the nasopharyngeal orifices of the ET were performed according to the study design (Fig. 1). A second sterile inflammation was induced in the first implanted ear one week before euthanasia, after 12 weeks. The degree of inflammatory reaction was evaluated by a daily check on the general health of the sheep and an endoscopic score of the pharyngeal orifice of the ET as described below.

### General anesthesia, implantation, and induction of inflammation

Implantation and induction of inflammation were performed under general anesthesia (GA) after sedation with Midazolam (0.2 mg/kg i.v.; Midazolam-ratiopharm® 5 mg/mL, Ratiopharm, Blaubeuren, Germany) and induction of GA with propofol (5–10 mg/kg i.v.; Propofol®-Lipuro 10 mg/mL, B. Braun Melsungen AG, Melsungen, Germany). For the maintenance of GA, Isoflurane (1.5–2.0% end-tidal inhalation; Isofluran CP 1 mL/mL, CP-Pharma, Burgdorf, Germany) was used. To prevent bleeding and to provide local anesthesia, pointed swabs wetted with Naphazolin 10 mL (Privin® 1 mg, Novartis, München, Germany) and Lidocaine 5 mL (Xylocain 2%, AstraZeneca GmbH, Wedel, Germany) were applied into both nostrils prior to the endoscopic approach.

Stenting was performed via a nasopharyngeal endoscopic approach to the ET. The stent, mounted on the catheter, was completely inserted into the epipharyngeal orifice of the ET via the working canal of a flexible broncho-fiberscope (Broncho-Fiberskop: 3.7 mm diameter, 1.5 mm working canal, 0° angle of view, 110° opening angle, 54 cm length, Karl Storz, Tuttlingen, Germany). The balloon of the catheter was inflated with physiologic saline solution and thus the stent expanded, using a pressure of 10 bar for two minutes. Afterward, the balloon was deflated, held in position for one minute and slowly extracted from the orifice of the ET. While the short stent (2.0 mm × 20 mm) stayed only in the cartilaginous part of the ET, the longer stent (2.75 mm × 26 mm) reached through the isthmus between the bony and cartilaginous parts where it became clamped during the balloon inflation process.

Inflammation was initiated using platelet activating factor 10^− 5^ mol/L (1-O-Hexadecyl-2-O-acetyl-sn-glycero-3-phosphocholine, Bachem GmbH, Weil am Rhein, Germany) and prostaglandin E_2_ 10^− 5^ mol/L (Prostaglandin E_2_, Sigma-Aldrich Chemie, Schnelldorf, Germany) delivered as a single treatment of 1 mL via a perforated balloon catheter at the time of first implantation in the unstented right ear and in a double concentration one week before euthanasia in the stented left ear. During the instillation of the fluid, no pressure occurred in the balloon or the ET, due to the perforation of the balloon. Postoperative pain management was provided by Carprofen 1.4 mg/kg i.v. (Rimadyl® 50 mg/mL, Pfizer, Berlin, Germany) and protection from bacterial inflammation performed with Benzylpenicillin-Dihydrostreptomycin 0.04 mg/kg s.c. (Veracin Comp®, Albrecht GmbH, Aulendorf, Germany).

### Health and endoscopic score

**Fig. 2:**
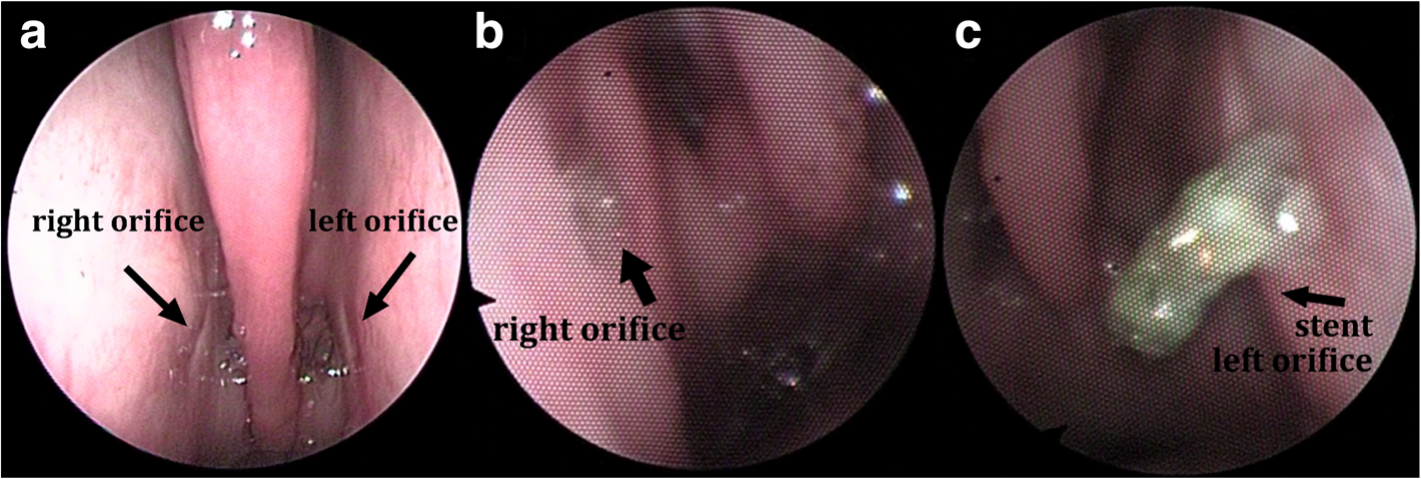
Endoscopic view of the ET for the evaluation of stent position and inflammation. Examples of (**a**) no [endoscopic score 1–3], (**b**) mild [score 4–6] and (**c**) moderate [score 7–9] inflammation in the nasopharyngeal orifice region are shown

To ensure the health of the animals, a daily check on their general health status and periodic endoscopic examinations of the ear and ET according to Fig. 1 were conducted. The health score according to Otto and Short [23] was modified, and included parameters such as breathing frequency, rumination, intake of food and water, head tilt or nasal discharge ranged from a score of zero (unaffected constitution) to seven (severely affected constitution) (see table provided as Additional file 1). The quality of mucus, the degree of opening of the nasopharyngeal orifice of the ET, inflammatory erythema, and swelling were accessed in the endoscopic score (Table 1). This score ranged from a maximum of 12 (severe inflammation) to a minimum of zero (no inflammation), with a score of zero to three categorized as non-inflammatory, from four to six as mild inflammation, from seven to nine as moderate inflammation, and from 10 to 12 as severe inflammation. Additionally, the visibility of the implanted stent in the proximity of the ET opening was documented. Examples of specific endoscopic images are presented in Fig. 2.

**Table 1:** Score for semi quantitative evaluation of the endoscopic images of the nasopharyngeal orifice of the ET

Criteria	Endoscopic score value			
	None	Mild	Moderate	Severe
Quality of mucus	0	1	2	3
Opening degree of n. orifice^a^	0	1	2	3
Inflammatory erythema	0	1	2	3
Inflammatory swelling	0	1	2	3

### Fixation, embedding, and staining procedure

After 12 weeks of implantation, sheep were euthanized under GA by an overdose of pentobarbital i.v. (Release® 300 mg/mL, WDT, Garbsen, Germany) before decapitation behind the second cervical vertebrae.

**Fig. 3:**
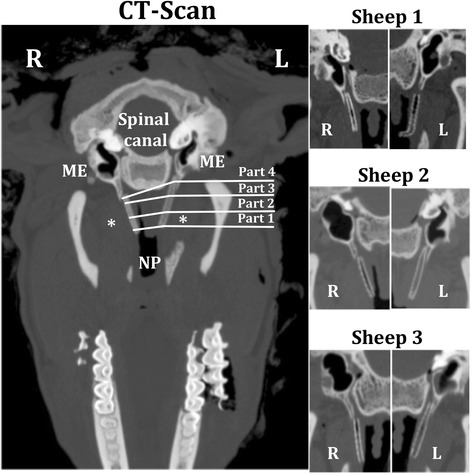
Coronal CT sections of both stents (*) in-situ for each sheep. Tympanic cavity (ME), nasopharynx (NP) and spinal canal were used for orientation and evaluation. The lines indicate the regions of histologic evaluation

Post-mortem, a spiral computed tomography (CT) scan was performed. On coronal reconstructions, the stent in its entire length as well as the middle ear and the external auditory canal were depicted (example shown in Fig. 3). The location of the stent and the degree of its obstruction (tissue or secretion) were determined. The middle ear, including the hypo-, meso- and epitympanum, was inspected regarding tissue formation and occurrence of effusion.

For histologic analysis, each ET with surrounding tissue was dissected, orienting on the nasopharyngeal opening and the opening to the middle ear, using a bone saw (FK23 bone saw, Bizerba, Balingen, Germany). Each specimen was washed in physiological saline solution (B. Braun Melsungen AG, Melsungen, Germany) and fixed in formalin (3.5%, pH 7.4; C. Roth, Karlsruhe, Germany) for two weeks. Prior to embedding in methylmethacrylate (MMA; Merck KGaA, Darmstadt, Germany) [24], dehydration of the specimen via an increasing ethanol series (70%, 80%, 90%, 100%; Merck) was performed. Each step was carried out overnight and finalized with MMA infiltration and polymerization in a water bath with increasing temperature (35 °C to 40 °C) for two to four days, depending on the status of polymerization. The excess MMA was removed with a plaster model trimmer (HSS 88, Wassermann Dental-Maschinen GmbH, Hamburg, Germany) until only the specimen remained. Specimen were cut into two halves and fixed on a specimen holder to cut slices of approx. 33 μm thickness with a saw microtome (Leica SP1600 ®, Leica Biosystems, Wetzlar, Germany), beginning in the middle of each ET and following the course of the ET in both directions. Additionally, slices of approx. 1 mm thickness were discarded at periodical intervals. Staining of the slices was performed with Alizarin red (Alizarin red S staining solution; Merck) and Methylene blue (Löffler’s Methylene blue solution; Merck). The slices were incubated for 45 s with Methylene blue on a heating plate (80 °C). After rinsing with distilled water, the slices were incubated with Alizarin red for 1.5 min. Drying in an incubator at 37 °C overnight followed an additional rinsing step. After staining, each slice was mounted on microscopic slides with Entellan®-new (Merck) and covered with cover slips.

### Histologic analysis and evaluation

Histologic analysis was performed with image editing software (NIS-Elements Imaging Software 4.20®, Nikon, Düsseldorf, Germany) after digitalization of the histologic slices under a microscope (SMZ1000 ®, Nikon, Düsseldorf, Germany, with a Nikon Digital Sight DS-Vi1 camera) at 2× magnification. In each set of slices, the end of the stent in the proximity of the nasopharynx was set as the starting point for the analysis while the end in the proximity of the middle ear opening was the endpoint of analysis. The position of the discarded slices was taken as reference to estimate the length of the tube and to divide the length of the stent in the tube into four parts. Part one represented the first third of the cartilaginous part of the ET and the beginning of the stent, following it from the nasopharyngeal opening. Part two adjoined it, representing the middle part, whereas part three covered the final third of the cartilaginous part of the tube in the direction of the middle ear. Due to the different lengths of the stents, the fourth part partially overlapped with the third part in the shorter stents but was positioned in the isthmus region for the longer stents, and revealed the ending of each stent. In each part, three representative concurrent slices were analyzed. In each slice, the lumen (L) of the ET, the amount of secretion (S) and tissue (T), and the total area of the ET, excluding the chondral and bony parts, gland tissue, or fat (ROI), were assessed. The free lumen (L_F_) was calculated by subtraction of the secretion from the lumen. Tissue, lumen, secretion and free lumen were given as percentages of the total ROI. The values of the three consecutive slices were averaged to obtain the results for a specific section of the ET. To calculate overall values averaged results were used.

**Fig. 4:**
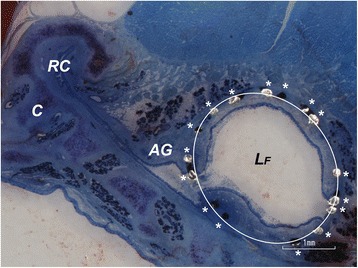
Slice of the ET with plotted ellipse illustrating the region of analysis of stent expansion. Depicted are Rüdinger’s safety canal (RC), auxiliary gap (AG), the tubal cartilage (C) and free lumen (L_F_) of the ET. An ellipse is depicted on the struts (*) indicating the position of the stent

An ellipse was positioned on the slices, such that the most visible struts of the stent were on or very close to the ellipse (Fig. 4). The stent diameter was determined via the stent area (the area of the ellipse) in the histological sections. The area was quantified in the slices for the four different parts of the ET and was compared to the references given by the manufacturer for each stent.

## Results

### Stent implantation and application of inflammatory mediators

In all three sheep, the endoscopic approach afforded a clear view on the pharyngeal orifice (Fig. 2), and implantation was easily completed on both sides. Furthermore, the insertion of the entire stent could be done without complication for both stent sizes. The application of inflammatory mediators using a perforated balloon was performed in the right ET prior to implantation and was not hampered by the previously implanted stent in the left ET at the end of the study.

### Health score and endoscopic score

During the entire period of the experiment, all three sheep displayed a health score of two or less from a maximum score of seven (see table in Additional file 2: summary of the specific findings for each sheep). Recurring periods of hot weather resulted in a 0.5 point increase due to the sheep’s enhanced breathing frequency. In sheep 3, serous nasal discharge was observed three times: two weeks prior to the first manipulation, during the week after the first general anesthesia before stent implantation and triggering of inflammation, and three weeks after stent implantation and first application of inflammatory mediators.

**Fig. 5:**
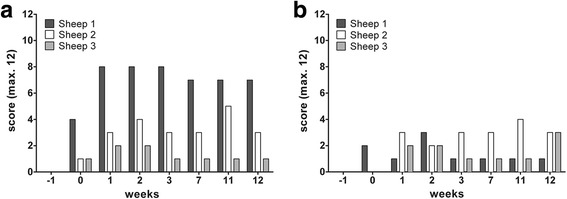
Recorded endoscopic score values. **a** Left (long stent) and (**b**) right (short stent) ET of each sheep under general anesthesia in the course of the experiment. In the first GA (week − 1) a score of zero was observed

The mean endoscopic score revealed no inflammatory signs for two of the short stents and one of the longer stents (Fig. 5). Mild inflammatory reactions occurred with one short and one long stent. A moderate reaction was detected for one long stent (Fig. 2c). Sporadic visibility in the pharyngeal orifice was apparent in all implanted stents. In sheep 1, the long stent was visible in all endoscopic examinations performed and in sheep 2, in four of the seven endoscopic examinations. In all three sheep an increase in secretion in the region of both ETs was detected relative to the first GA before implantation. Neither in the health score nor in the endoscopic score (Fig. 5), severe inflammation was detected in all three sheep showing a temporal connection to the instilled inflammatory mediators.

### CT scans

Coronal reconstructions (Fig. 3) demonstrated ventilated middle ears with few or minimal accumulations of secretion and limited soft tissue formations. The CT scans displayed air filling with proximal obstruction in all short stents and one long stents (sheep 1). In contrast to this, the remaining longer stents appeared to be fully obstructed. All stents were located in the chondral part of the ET. In sheep 1, the long stent showed a proximal dislocation in the direction of the nasopharynx, and in sheep 2 the shorter stent was located directly in the nasopharyngeal orifice of the ET. Both accurately positioned longer stents showed narrowing in the bony part and isthmus of the ET.

### Histologic analysis

**Fig. 6:**
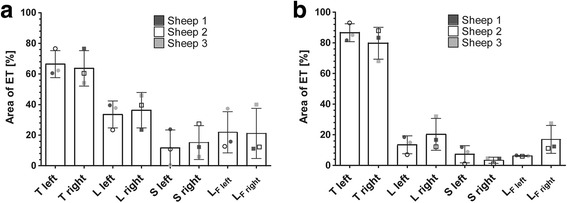
Areas of tissue occurrence (T), lumen (L), secretion (S) and free lumen (L_F_). Depicted are parts 1 (**a**) and 4 (**b**) of the ET for both stents (compare Fig. 3). The total area of the ET (ROI) was set as 100%

Tissue formation was assessed in the histologic analysis. The ET stented with the 2.75 mm × 26 mm sized implant covered on average an area (ROI) of 10.2 ± 2 mm^2^ with 6.6 ± 2.2 mm^2^ tissue (T) and 3.6 ± 0.3 mm^2^ lumen (L). The lumen was filled with 1.2 ± 1 mm^2^ secretion (S), leaving a free lumen (L_F_) of 2.4 ± 1.1 mm^2^. The ET implanted with the smaller stent showed a ROI of 7.2 ± 2.2 mm^2^ with 4.9 ± 1.8 mm^2^ tissue and 2.4 ± 0.8 mm^2^ lumen, with the latter filled with 0.8 ± 0.4 mm^2^ secretion, leaving a free lumen of 1.5 ± 0.7 mm^2^. The values for each sheep are summarized in Additional file 2. When evaluating the tissue in the different parts (1 to 4) of the tube, in both ETs an increase in tissue as well as a decrease in lumen, secretion and free lumen was detected from the nasopharynx (part 1) to the middle ear (part 4) (Fig. 6), with only minor differences among parts 1 to 3 (not shown).

### Stent dimensions and lumen-tissue interface

**Fig. 7:**
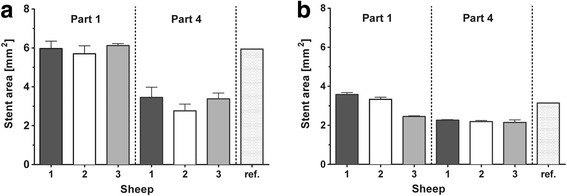
Mean stent area. Parts 1 and 4 (compare Fig. 3) of the implanted 2.75 mm × 26 mm (**a**) and 2.0 mm × 20 mm (**b**) stents with the calculated reference for each stent size. The data are shown as mean + SD

**Fig. 8:**
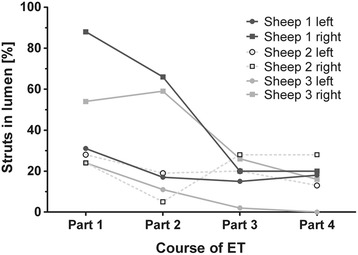
Percentage of struts not covered by tissue. Depicted from the nasopharynx (part 1) to the middle ear (part 4) for both types of stents

In general, both implanted stent sizes were expanded up to their nominal diameter (Additional file 2). In part one, close to the nasopharyngeal opening, five of the six stents were expanded almost to their full diameter. In contrast to this, all six stents displayed a smaller degree of expansion in part four, at or close to the bony isthmus (Fig. 7). The struts of both stents generally maintained their circular arrangement and could be detected in the sub-mucosal layer. However, in sheep 3, the struts in part 4 appeared to be deeper in the tissue, leaving the mucosal layer and becoming embedded in muscle and gland tissue. The general percentage of struts lying free in the lumen ranged from 6.5% to 43%, and was generally higher in the right ETs, i.e., the smaller stent (Fig. 8).

**Fig. 9:**
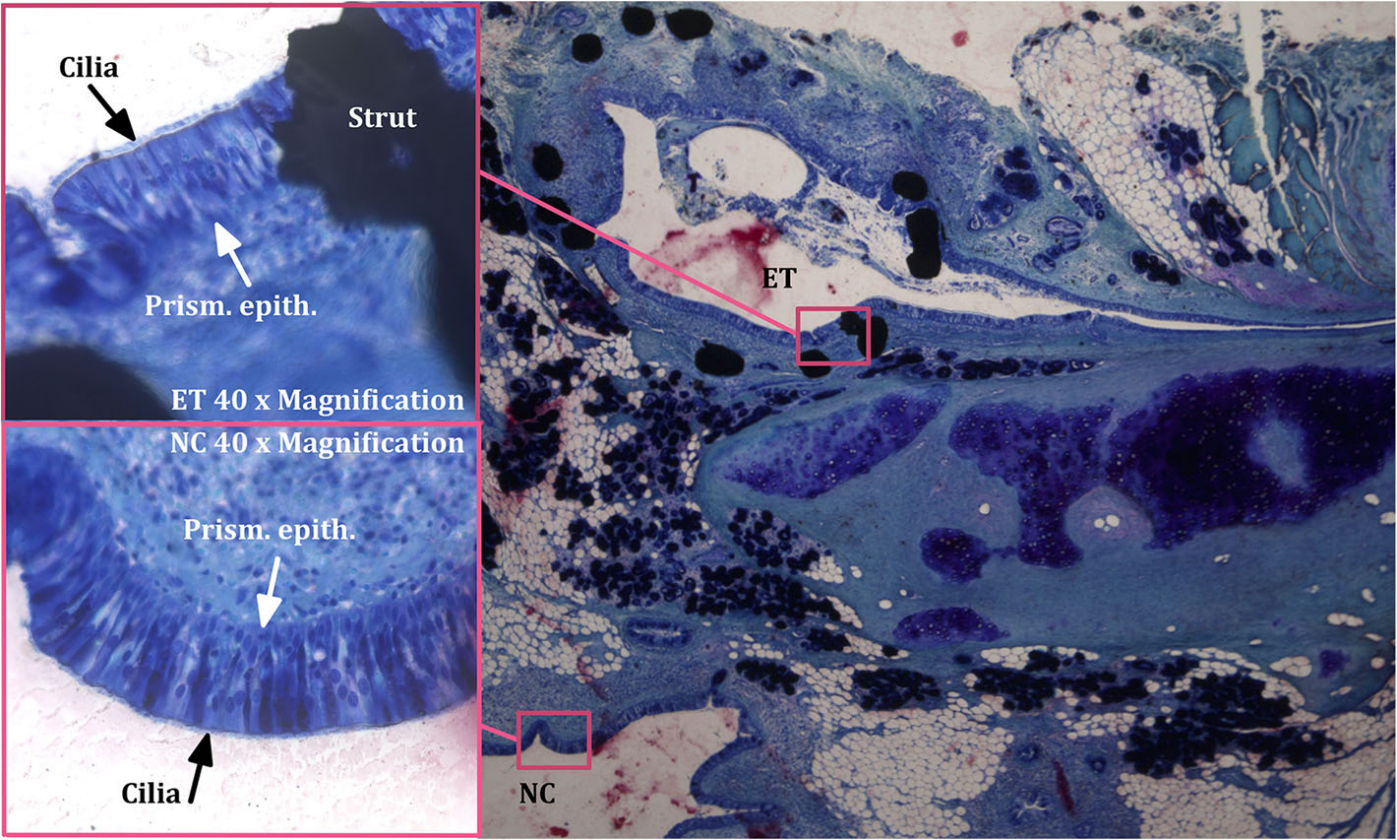
Interface between mucosa and lumen. Illustrated are the Eustachian tube (ET) and the nasal cavity (NC) of sheep 3 (right ET, part 4) showing prismatic epithelium (Prism. epith.) topped with cilia at 2× magnification

The first mucosal layer on the interface between mucosa and lumen of the ET consisted in each ET of prismatic epithelium. The luminal side of this epithelium was covered with cilia, which could also be detected in the respiratory epithelium of the nasopharynx (Fig. 9). In sheep 3, both ETs showed moderate signs of mucosal detachment and autolysis, yet fragments of prismatic epithelium and cilia could be detected as well in both ETs in this sheep.

## Discussion

Improvement of middle ear ventilation via ventilation tubes in the tympanic membrane causes tympanic membrane perforation and may create new pathways for entry of pathogenic microorganisms from the external auditory canal. If used repeatedly, atrophic scarring of the tympanic membrane, myringo- and tympanosclerosis, tympanic membrane retraction, persistent perforation, or granulation tissue formation become apparent with an incidence of 51% [25, 26]. Few therapeutic approaches utilize the ET itself with a nasopharyngeal approach for the treatment of chronic otitis media and ETD. Stenting the ET could improve ventilation of the middle ear and clearance of secretion through the ET itself, preventing tympanic membrane perforation and over the long term, middle ear destruction.

Thus, the primary aim of the present study was to investigate stent implantation from the nasopharynx into the ET in-vivo in blackface sheep, and to evaluate possible body reactions. The secondary aim was to develop a model of induced aseptic OME, as the need for an intervention only arises when the function of ET is impaired.

The application of inflammatory mediators into the middle ear of sheep via the ET was easily performed. As described in human practice [14], small amounts of fluid (one to two milliliters) could be instilled in the sheep through the working channel of the flexible endoscope using a catheter perforated at its tip. The amount of fluid deposited in the middle ear was difficult to determine precisely, because of an apparent efflux from the nasopharyngeal orifice. However, a considerable amount of fluid and therefore inflammatory mediators potentially reached the middle ear and ET, resulting in the physiological absorption from middle ear and tubal mucosa. In contrast to the findings in the chinchilla model of induced OME triggered by inflammatory mediators applied through the bulla [21], no signs of moderate or severe inflammation with a temporal connection to the instillation of PAF and prostaglandin E_2_ were observed. The effective dosage of PAF necessary to induce middle ear effusion in mongrel dogs is reported to be between 10^− 7^ mol/L and 10^− 6^ mol/L [27], indicating that the dosage of 10^− 5^ mol/L first used in the current study and especially the doubled concentration in the second application, should have been sufficient to trigger the desired effect. According to a study investigating the inflammatory potency of PAF, induced OME lasts for up to 14 days in chinchillas due to the initiation of an inflammatory cascade, although the initial inflammatory mediator was already cleared from the body [22]. However, the peak of the inflammatory reaction was suggested to occur on day 4 post-inflammation [21]. For these reasons we expected the inflammation in the sheep to peak during the first week after application, and to still be visible on day seven. Due to species restraints regarding the frequency of anesthesia (limited to once a week), the follow up was performed on day seven after the instillation of PAF and prostaglandin E_2_. Unfortunately, use of the tympanometry method in sheep [28] was not available at the time the examination was performed, which would have facilitated monitoring of middle ear effusion or negative middle ear pressure [3] at shorter intervals. Therefore, we can only speculate about the reasons for missing inflammatory signs related to the application of the substances. Amongst the possible reasons are (i) clearance of all the inflammatory mediators through the ET, (ii) a lower susceptibility of the sheep to the substances, or (iii) a reaction that does not present symptoms detectable with the applied methods one week after instillation. Even though we failed in inducing detectable inflammation, this should have no impact on the other results of this study.

Stent implantation in-vivo was shown to be quite feasible, as in the cadaver (compare [18]). All sheep were healthy during the experiment and the sporadic incidences of serous secretion in sheep 3 may be explained by dust particles in hay and straw, irritating the mucous membrane. This phenomenon had already been observed before stent implantation, and in veterinary practice it is common in a variety of species living in dusty environments.

During stent implantation, no erosion of the carotid artery was observed. This was expected, as the same pressure recommended for balloon dilatation was used, the diameters of both stents were smaller than that of the balloon, and balloon dilatation does not have adverse effects on the bony part of the ET [29].

Five of the six inserted stents stayed in position where implanted. Fixation of the shorter stents only in the cartilaginous part of the ET was successful in all three instances, and fixation of the larger stent in the isthmus was successful in two of three cases. In one sheep, shortly after implantation, the stent migrated in the direction of the nasopharynx, eventually reaching into the nasopharynx. However, the stent stayed in this position for the rest of the observation period. According to these findings, fixation at the isthmus may not be required. This fact is important particularly because consequences of stent placement in the isthmus seem to include a narrower stent diameter in the vicinity of the bony isthmus and local ET distortion with further impaired clearance function. This may have led to difficulty with mucus transport and resulted in the obstruction of the stents and finally the ET itself, although secretion was seen in all correctly positioned 26 mm stents on CT scans. Even though no signs of increased inflammation were found in the histology of these stents, it remains unclear whether the application of the inflammatory mediators might have contributed to the obstruction of the long stents at the isthmus. The single dislocated stent had a position closer to that of the 20 mm stents and showed similar ventilation with a smaller degree of narrowing compared to the other 26 mm stents.

In general, mild inflammation was detected in the endoscopic images of the ET orifice at the nasopharynx as well as moderate connective tissue encapsulation in the histologic slices. Additionally, rare signs of inflammation and minimal accumulation of secretion were seen in the middle ears. As the entrance of pathogenic microorganisms is limited to the pharynx and prevention of ascending infections to the middle ear is one of the main tasks of the ET [30], the protective function appears to be maintained, at least over the observation period of three months. However, dislocation of the stent should be avoided, as direct contact of the stent with bacterial flora in the nasopharynx might have led to an increase of purulent secretion and inflammation. Further, the extruded part of the stent is expected to cause continuous mechanical irritation during all epipharyngeal movements. Nonetheless, the middle ear itself appeared to be as unaffected as in the other sheep.

With the stents in this study we might have created a permanently open ET. A permanently open ET is a symptom of a patulous ET [31], allowing sounds of speech and nasopharyngeal sounds (autophony), reflux from the gastrointestinal tract [32], and pathogenic microorganisms [3] to ascend into the middle ear, leading to sickness of the patient as well as mucosal irritation and infections. Thus, migration into the nasopharynx and direct placement of the stent in the nasopharyngeal opening of the ET should be avoided. Ideally, a stent would be positioned in the ET such that it facilitates opening, but does not cause a permanently open ET.

The free lumen was calculated to be about 20% in both ET, which is 2.35 mm^2^ in the cross section of the larger and 1.5 mm^2^ in the smaller stent. The isthmus, the narrowest portion of the ET, measures 1 mm in width × 3.5 mm in height in the blackface sheep [18] and 1 mm × 2 mm [4] in humans, implying that the detected free lumen should be enough to promote permanent ventilation as it is described for Rüdinger’s safety canal in the tubal cartilage, which measures only 0.4 to 0.5 mm [33]. In contrast, a stent diameter of 1.5 mm was not sufficient to maintain transport of secretion in an earlier study [8], thus, the obstruction with secretion in the longer stents might be caused by the narrowing of the stent diameter at the isthmus, explaining why the dislocated larger stent did not show total obstruction.

Additionally, the movement of the auxiliary gap, i.e. the movable space below the safety canal, which opens only in process of swallowing or yawning, is hampered by the ingrown stent (Fig. 4), limiting its opening diameter to the stent diameter. This part of the ET in humans physiologically provides an opening diameter of up to 6–10 mm [18], ensuring the clearance [33]. This diameter in sheep was reduced by the ingrown stent to 2.75 mm or less.

In coronary vessels, an overgrowth of the vascular graft with intima is desirable to prevent thrombus formation and re-establish a smooth surface, ideally mimicking the original surface of the endothelium [34]. This overgrowth was observed in all specimens in the current study, leaving only an average of 25.4% of the struts in the lumen. Furthermore, the appearance of ciliated epithelium and prismatic cells, in the epithelium of the nasopharynx and usually as well in the ET [35], indicates a reparation of the mucosal layer traumatized by the insertion of the stent and therefore closure of the tissue-lumen interface. Thus, the stent is incorporated and fixed in its position. This phenomenon may facilitate the clearance, but may also bear the risk of excessive growth of tissue. Finally, explantation of the stent is not advisable because surgical removal would be accompanied by the removal of mucosa, which may cause the ET to coalesce in the process of healing.

## Conclusion

Application of fluids into the ET and middle ear of blackface sheep was feasible, but the reaction of the inflammatory mediators was not as extensive as expected. The non-traumatizing and minimally invasive procedure of stenting the ET was successfully transferred from cadaver studies to in-vivo application without complications. The stent was well tolerated by the sheep and did not hamper ventilation of the middle ear or clearance of the ET. Regarding the design of the stent, it seems to be sufficient to place it only in the cartilaginous part of the ET, but the length and/or positioning should be adjusted to prevent a permanently open, and therefore patulous, ET.

## Additional files

Additional file 1: Health score as used in the study (modified from Otto and Short 1998). (DOCX 21 kb)

Additional file 2: Summary of the specific findings for each sheep. (DOCX 39 kb)

## Abbreviations

CT: Computed tomography
ET: Eustachian tube
ETD: Eustachian tube dysfunction
GA: General anesthesia
MMA: Methylmethacrylate
OME: Otitis media with effusion
PAF: Platelet activating factor
