# Supplementary material for: Stenting the Eustachian tube to treat chronic otitis media - a feasibility study in sheep
Source: Head Face Med. 2018 May 4;14:8. doi: 10.1186/s13005-018-0165-5 (PMC5935938; doi:10.1186/s13005-018-0165-5)
Supplement: Supplementary file 1 — Health score as used in the study (modified from Otto and Short 1998). (DOCX 21 kb) [file 13005_2018_165_MOESM1_ESM.docx]

**Table additional file 1**

Health score as used in the study (modified from Otto and Short 1998)

| **Category** | **Observed behaviour** | **Score** |
| --- | --- | --- |
| **Intake of food and water** | Usual food and water intake, usual rumination | **0** |
|  | Only treats and moderate rumination | **1** |
|  | No feeding and water intake, no rumination | **2** |
| **Behaviour and facial expression** | Interested in surroundings, nibbles straw, head is carried upright and straight | **0** |
|  | Depressed, tired, sporadic moderate head tilt and /or head shaking | **1** |
|  | Flehming, absent staring, permanent head tilt and / or head shaking | **2** |
| **Breathing frequency *** | Up to 20 breaths /min | **0** |
|  | 25-30% increase from reference value | **0.5** |
|  | More than 50% increase from reference value | **1** |
| **Additional anomalies** | None | **0** |
|  | Increased body temperature, serous nasal discharge | **1** |
|  | Fever, purulent or bloody nasal discharge | **2** |
| **Maximum score** |  | **7** |

* breathing frequency can be enhanced by several reasons including high temperatures – therefore this category should not have the same influence as the other categories
