# Supplementary material for: Stenting the Eustachian tube to treat chronic otitis media - a feasibility study in sheep
Source: Head Face Med. 2018 May 4;14:8. doi: 10.1186/s13005-018-0165-5 (PMC5935938; doi:10.1186/s13005-018-0165-5)
Supplement: Supplementary file 2 — Summary of the specific findings for each sheep. (DOCX 39 kb) [file 13005_2018_165_MOESM2_ESM.docx]

**Table additional file 2**

Summary of the specific findings for each sheep.

|  | ***Sheep 1*** | | ***Sheep 2*** | | ***Sheep 3*** | |
| --- | --- | --- | --- | --- | --- | --- |
| **Health score (0-7)** | **Mean:** 0.16 **Max:** 0.5 **Min:** 0 | | **Mean:** 0.14 **Max:** 2 **Min**: 0 | | **Mean:** 0.19 **Max:** 0.5 **Min:** 0 | |
| **Stent size** | **2.75 mm x 26 mm (left)** | **2.0 mm x 20 mm (right)** | **2.75 mm x 26 mm (left)** | **2.0 mm x 20 mm (right)** | **2.75 mm x 26 mm (left)** | **2.0 mm x 20 mm (right)** |
| **Implantation** | **Insertion:** possible  **Position after implantation:** visible in prox. orifice | **Insertion:** possible  **Position after implantation:** behind prox. orifice | **Insertion:** possible  **Position after implantation:** behind prox. orifice | **Insertion:** possible  **Position after implantation:** behind prox. orifice | **Insertion:** possible  **Position after implantation:** behind prox. orifice | **Insertion:** possible  **Position after implantation:** behind prox. orifice |
| **Endoluminal application of inflammatory mediators** | **With stent:**  Possible, fluid leakage from prox. orifice | **Without stent:**  Possible, fluid leakage from prox. orifice | **With stent:**  Possible, fluid leakage from prox. orifice | **Without stent:**  Possible, fluid leakage from prox. orifice | **With stent:**  Possible, fluid leakage from prox. orifice | **Without stent:**  Possible, fluid leakage from prox. orifice |
| **Endoscopic score (0-12)** | **Mean:** 6.13  **Max:** 8.0  **Min:** 0.0  **Stent visible:** 7/7 | **Mean:** 1.25  **Max:** 3.0  **Min:** 0.0  **Stent visible:** 0/7 | **Mean:** 2.75  **Max:** 5.0  **Min:** 0.0  **Stent visible:** 4/7 | **Mean:** 2.25  **Max:** 4.0  **Min:** 0.0  **Stent visible:** 3/7 | **Mean:** 1.13  **Max:** 2.0  **Min:** 0.0  **Stent visible:** 2/7 | **Mean:** 1.25  **Max:** 3.0  **Min:** 0.0  **Stent visible:** 2/7 |
| **CT Scan** | **Stent position:** Prox. dislocated  **Stent lumen:**  Air-filled, prox. obstructed  **H-Tymp:**  Minimal accumulation of secretion, air-filled  **M & E-Tymp:**  Minimal soft tissue | **Stent position:** Cartilaginous part  **Stent lumen:**  air-filled, prox. obstructed  **H-Tymp:**  Small accumulation of secretion, air-filled  **M & E-Tymp:**  Few soft tissue | **Stent position:** Cartilaginous part  **Stent lumen:**  Obstructed  **H-Tymp:**  Air-filled  **M & E-Tymp:**  Minimal soft tissue | **Stent position:** Cartilaginous part  till prox. opening  **Stent lumen:**  Air-filled, prox. obstructed  **H-Tymp:**  Air-filled  **M & E-Tymp.:**  Minimal soft tissue | **Stent position:** Cartilaginous part  **Stent lumen:**  Obstructed  **H-Tymp:**  Small accumulation of secretion, few soft tissue  **M & E-Tymp:**  Few soft tissue | **Stent position:** Cartilaginous part  **Stent lumen:**  Air-filled, prox. obstructed  **H-Tymp:**  Air-filled  **M & E-Tymp:**  Minimal soft tissue |
| **Position during experiment** | Prox. dislocation of about 25% of length | As inserted | As inserted | Slightly visible | As inserted | As inserted |
| **Histological analysis (all four parts)** | **ROI:** 100% / 12.35 mm^2^  **T:** 73.44% / 9.07 mm^2^  **L:** 26.56% / 3.28 mm^2^ **S:** 11.84% / 1.46 mm^2^  **L_F_:** 14.72% / 1.82 mm^2^ | **ROI:** 100% / 9.82 mm^2^  **T:** 69.35% / 6.81 mm^2^  **L:** 30.65% / 3.01 mm^2^ **S:** 13.26% / 1.3 mm^2^  **L_F_:** 17.39% / 1.71 mm^2^ | **ROI:** 100% / 8.41 mm^2^  **T:** 55.66% / 4 .68 mm^2^  **L:** 44.34% / 3.73 mm^2^ **S:** 25.23% / 2.12 mm^2^  **L_F_:** 19.11% / 1.61 mm^2^ | **ROI:** 100% / 6.04 mm^2^  **T:** 75.10% / 4.54 mm^2^  **L:** 24.90% / 1.50 mm^2^ **S:** 13.55% / 0.82 mm^2^  **L_F_:** 11.35% / 0.69 mm^2^ | **ROI:** 100% / 9.76 mm^2^  **T:** 61.65% / 6.02 mm^2^  **L:** 38.35% / 3.74 mm^2^ **S:** 0.94% / 0.09 mm^2^  **L_F_:** 37.41% / 3.65 mm^2^ | **ROI:** 100% / 5.90 mm^2^  **T:** 56.77% / 3.35 mm^2^  **L:** 43.23% / 2.55 mm^2^ **S:** 7.51% / 0.44 mm^2^  **L_F_:** 35.72% / 2.11 mm^2^ |
| **Stent dimensions (mm^2^)**  **Ref. left: 5.94 mm^2^**  **Ref. right: 3.14 mm^2^** | **Mean:** 5.22  **Max:** 6.37  **Min:** 2.85 | **Mean:** 2.56  **Max:** 3.70  **Min:** 1.92 | **Mean:** 4.18  **Max:** 6.16  **Min:** 2.57 | **Mean:** 2.98  **Max:** 3.56  **Min:** 2.13 | **Mean:** 5.36  **Max:** 6.55  **Min:** 3.03 | **Mean:** 2.37  **Max:** 2.63  **Min:** 2.01 |
| **Interface mucosa & lumen** | **Ciliated epith.:** yes  **Prismatic epith.:** yes | **Ciliated epith.:** yes  **Prismatic epith.:** yes | **Ciliated epith.:** yes  **Prismatic epith.:** yes | **Ciliated epith.:** yes  **Prismatic epith.:** yes | **Ciliated epith.:** yes  **Prismatic epith.:** yes | **Ciliated epith.:** yes  **Prismatic epith.:** yes |
| **Struts in lumen (%)** | **17.5** | **43** | **19.5** | **26** | **6.5** | **40** |

Distal = in direction of middle ear; Prox. = Proximal = in direction of nasal cavities; M & E-Tymp = meso- and epitympanon; H-Tymp. = hypotympanon; Epith. = epithelium; ROI = entire Eustachian tube, excluding bony and chondral parts; L = lumen; S = secretion; T = tissue; L_F_ = free lumen
